# Rapid Prototyping of 3D Biochips for Cell Motility Studies Using Two-Photon Polymerization

**DOI:** 10.3389/fbioe.2021.664094

**Published:** 2021-04-13

**Authors:** Federico Sala, Carlotta Ficorella, Rebeca Martínez Vázquez, Hannah Marie Eichholz, Josef A. Käs, Roberto Osellame

**Affiliations:** ^1^Department of Physics, Politecnico di Milano, Milan, Italy; ^2^Istituto di Fotonica e Nanotecnologie, Consiglio Nazionale delle Ricerche, Milan, Italy; ^3^Peter Debye Institute for Soft Matter Physics, University of Leipzig, Leipzig, Germany

**Keywords:** femtosecond laser microfabrication, two-photon polymerization, lab-on-a-chip, neuronal cell, cell migration

## Abstract

The study of cellular migration dynamics and strategies plays a relevant role in the understanding of both physiological and pathological processes. An important example could be the link between cancer cell motility and tumor evolution into metastatic stage. These strategies can be strongly influenced by the extracellular environment and the consequent mechanical constrains. In this framework, the possibility to study the behavior of single cells when subject to specific topological constraints could be an important tool in the hands of biologists. Two-photon polymerization is a sub-micrometric additive manufacturing technique that allows the fabrication of 3D structures in biocompatible resins, enabling the realization of *ad hoc* biochips for cell motility analyses, providing different types of mechanical stimuli. In our work, we present a new strategy for the realization of multilayer microfluidic lab-on-a-chip constructs for the study of cell motility which guarantees complete optical accessibility and the possibility to freely shape the migration area, to tailor it to the requirements of the specific cell type or experiment. The device includes a series of micro-constrictions that induce different types of mechanical stress on the cells during their migration. We show the realization of different possible geometries, in order to prove the versatility of the technique. As a proof of concept, we present the use of one of these devices for the study of the motility of murine neuronal cancer cells under high physical confinement, highlighting their peculiar migration mechanisms.

## Introduction

Additive manufacturing, also known as “3D printing,” is nowadays a standard technique for the realization of 3D objects. Its versatility in geometry design and the possibility to use different materials, from plastics to metals, make it ideal for industrial prototyping or custom design fabrications. The sub-micron counterpart of 3D printing is direct laser writing, also called two-photon polymerization (2PP) (Lee et al., 2006). This technique makes use of ultrashort laser pulses, usually in the femtosecond regime, to trigger the polymerization of specific liquid-phase photosensitive resists by multiphoton absorption. The use of this non-linear absorption process guarantees that only within the focal volume (voxel) of the laser beam the energy intensity is sufficient to induce resist solidification, without causing undesired photopolymerization. Translating the voxel inside the material permits the drawing of 3D geometries with arbitrary design. Afterward the unpolymerized resist can be removed with appropriate solvents, leaving behind only the laser-written structure. The minimum feature dimension is ultimately given by the size of the multiphoton absorption voxel and it can be tailored down to a few tens of nanometers (Emons et al., 2012).

In the last two decades, 2PP has been used for the realization of several types of microstructures, from micro-optics (Guo et al., 2006; Liberale et al., 2010) to microfluidic elements (Lim et al., 2011; Amato et al., 2012), passing through micromechanical structures (Jonušauskas et al., 2019). Thanks to the combination of small feature size and biocompatibility of some resists, one of the most interesting application is the realization of structures for biological studies (Nguyen and Narayan, 2017). Indeed, 2PP is ideally suited for creating biomimetic microenviroments that reproduce the complexity and heterogeneity of tissues.

The manufacturing of 3D supports to mimic the natural cellular environment is a flourishing field of application of 2PP, for example in the growth of stem cells for regenerative medicine applications (Raimondi et al., 2012). It is known from biophysical studies that the cells “feel” their microenvironment and that external mechanical factors impact their gene expression through mechanotransduction, without the influence of other biochemical factors (Raimondi, 2006). Consequently, the control of the extracellular matrix during growth has become an effective technique to prevent or induce cellular proliferation or differentiation. The use of 3D scaffolds for cell culture showed an increased motility of the cells toward the 3D structures (Käpylä et al., 2012; Raimondi et al., 2013), and a cell density comparable with flat surface colonies, highlighting not only that the structures do not hinder cellular proliferation, but also that cells are prone to grow on 3D structures. Furthermore, probably due to a reduced stress of the cells (if compared to 2D culture) the multipotency of stem cells is maintained during the growth on 3D scaffolds (Raimondi et al., 2014). These results were possible thanks to the investigation of several structures with different geometries and dimensions, tailored for the specific study and employed cell type, which is granted by the 2PP fabrication capabilities. The more recent developments in 2PP laser writing technology allowed the fabrication of far more complex scaffold geometries, for the study of many different cell types and their behavior when cultured on these 3D structures (Hippler et al., 2019). For example the dimension and the shape of the scaffold pores can influence not only the proliferation of the cells, but also their invasiveness. Studies on the stiffness of the scaffold material demonstrate that also this parameter could influence cellular response to the structure, showing for instance that tumoral cells prefer “softer” materials. Scaffolds with a fully custom 3D geometry, with empty pillars for cell adhesion and microtubes as connections between them, were used by Fendler et al. (2019) to guide the growth of neuronal cells and of their axons into a pre-determined neuronal circuit, opening the possibility to singularly investigate the 3D connection between neurons with patch-clamp experiments. A different evolution of the technique is linked to the composition of the employed photoresist. Multi-material biofunctionalized scaffolds (Hippler et al., 2019) were used to investigate protein affinity and cell adhesion properties, whereas biodegradable polymers are studied in order to create structures that can be absorbed by the tissues with time. An interesting example is presented by Accardo et al. (2018) combining an hydrogel matrix with a photoinitiator in order to apply direct laser writing on this new class of materials. Hydrogels present many interesting properties as low stiffness and optical transparency. In the study, the team cultivated neuronal cells onto a woodpile hydrogel scaffold, obtaining an efficient growth a of ramified neuronal network also inside the structure itself.

Another physiological cell process that is strongly affected by extracellular factors is cell migration in dense tissues, which constitutes one of the main processes in the physiology and pathology of multicellular organisms (Charras and Sahai, 2014; Yamada and Sixt, 2019). Cell motility is activated by external signals (chemical or mechanical); due to the broad variability of the process, a complete understanding of migration mechanism, in particular in 3D in dense environments, is still needed. Among others, the physical properties of the microenvironment, for example, the topology and confinement (Ilina et al., 2020), are known to influence the migration of cells, conditioning characteristics such as migration velocity or even migration strategy. A standard tool used for these types of experiments is the Boyden chamber (Albini et al., 1987), where cells migrate through a porous filter due to a chemical gradient between an upper and a lower chamber. The main drawbacks of this approach are that no imaging is possible during the migration and that it only permits the use of randomly organized polymer networks as an extracellular matrix. A recent alternative that allows real time monitoring of the cells in motion is based on microfluidic approaches. The basic scheme is made of a microchannel, with dimensions much smaller than the cell under study, which is used to monitor cell migration in a confined environment. Real tissues are packed close to volume fraction one (Grosser et al., 2021) and thus confinement effects play a major role. These constrictions, whose smaller features are around 1–5 μm can be realized with different techniques. The most common is the PDMS replication of master molds realized with photolithographic techniques (Denais et al., 2016). However, PDMS devices require complex multilayer composition approaches to realize channels with 3D variable geometries and are hardly reusable. An alternative is the use of direct laser writing to realize directly the desired geometry with the required micron-size resolution. Hjortø et al. (2015) realized some micrometric constrictions using 2PP inside a commercial plastic microfluidic device and optimized the geometry to guarantee a uniform chemical gradient for the chemotaxis. Sima et al. (2018) used direct laser writing to realize a Foturan glass microfluidic device and a set of polymeric micro-constrictions directly inside the microchannel, for the analysis of prostate cancer cells. They show the possibility to change the constriction length and dimension by simply modifying the photoresist irradiation geometry. However, the residual wall roughness (around 25 nm RMS) and the glass layer thickness introduce optical aberrations during imaging, thus compromising the possibility to optically monitor cell migration. In more recent work (Sima et al., 2020), the same group improved the structuring process in order to realize the micro-constrictions directly in glass. They reduced the bottom glass layer thickness and shrank down the constrictions dimension to less than 1 μm, still maintaining glass as the only employed material. This level of precision in Foturan glass microstructuring is indeed at the state of the art, but the nature of the process, driven by surface tension relaxation, limits the possible geometries achievable with this technique. In this framework, it is predictable that a technology which will allow a fast and easy tailoring of the migration environment topology, while still allowing the optical monitoring of cell behavior, will pave the way to unprecedented *in vitro* cell migration studies.

In our work we present a novel strategy to implement a microfluidic platform for cell motility studies that combines the *ad hoc* fabrication possibilities of 2PP with excellent optical accessibility. Our approach is to realize a multilayer structure composed by a top thick glass layer with reservoirs for samples deposition, a bottom thin optical-quality glass layer for inverted microscope imaging and an intermediate polymeric layer for the realization of arbitrarily shaped constrictions. Using this combined approach, we are able to realize microfluidic devices with different environment topologies for studying cell motility. The observation area of cell migration is optically accessible, allowing to acquire high-resolution fluorescence images of the migrating cells for several hours as needed for vital cell tracking. As a proof of concept, we use here one of the topologies to study murine neuronal cancer tumor cell behavior in high physical confinement. This study shows the high invasiveness of this type of cells through small pores and the dynamic of their migration.

## Materials and Methods

### Multilayer Device Fabrication Process

The design of the device consists of two 2 mm × 3.4 mm sized reservoirs connected by a migration region containing several micrometer sized funnel-like structures (constrictions) (see Supplementary Figure 1). The two reservoirs are dedicated one to the cell growth and the other to collecting migrating cells as well as containing chemoattractant if needed. They should be able to host a volume of growth media sufficient to carry out several hours of experiments before complete evaporation. The constrictions may have different shapes and dimensions, but they should be smaller than the typical size of the cell family under analysis. In this way the cells will have to squeeze and change their morphology to pass through the funnel and reach the second reservoir. All the remaining edges of the reservoirs should be completely sealed, in order to allow the cell passage only through the constrictions.

The funnel structures have a lateral dimension of about 100 μm at the input and minimum aperture between 10 and 5 μm at the output, which is smaller than the average size of animal cells (10–20 μm). Because they have to be realized in a biocompatible material and with a precise and freely designable geometry, 2PP is ideal for the realization of such structures, both in terms of feature size and possibility to easily realize different geometries for carrying out comparative studies using constrictions with different dimensions and shapes. On the other hand, realizing a whole microfluidic device with millimeter sized reservoir is rather challenging and time-consuming with this technique. A way to circumvent this problem is to realize the reservoirs and microchannel with other techniques and then laser-write the polymeric structures directly inside it (Wu et al., 2014). Glass surface microstructuring techniques, such as micro-drilling or laser ablation, do not allow the realization of embedded microchannels; thus a second step of channel sealing is required for the micro-constriction realization. Furthermore, the residual surface quality with these techniques tends to be quite poor and could influence cell adhesion during motility experiments. Femtosecond laser irradiation of fused silica glass, combined with chemical etching (FLICE) (Osellame et al., 2011), allows the realization of buried microchannels with millimeter length and feature sizes of few microns (Kiyama et al., 2009). However, the residual surface roughness [around 60 nm RMS in the case of fused silica substrate (Ho et al., 2012; Di Donato et al., 2016)] could affect the image quality through the microchannel walls.

**FIGURE 1:**
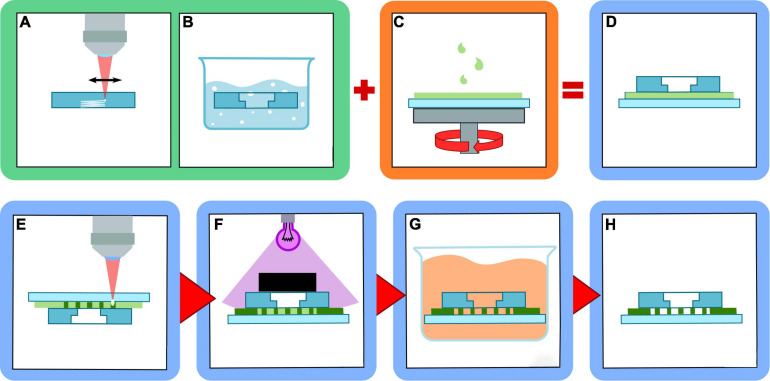
Scheme of multilayer device fabrication procedure. **(A)** fs-laser irradiation of reservoirs inside fused silica layer; **(B)** selective etching of the irradiated structures in HF solution; **(C)** resist spin-coating on the glass coverslip; **(D)** multilayer structure assembling; **(E)** micro-constrictions fabrication with 2PP; **(F)** device sealing with UV illumination and masking; **(G)** unpolymerized resist removal by solvent bath; **(H)** final device assembled.

We decided to use a composite-device approach that is schematically represented in Figure 1. Firstly, the millimetric sized reservoirs are realized with FLICE technique on a thick fused silica substrate. They are then assembled in a multilayer structure with a thin film of photoresist and a thin coverglass. Finally, the 2PP constriction are then realized in the intermediate resist layer. The three steps are separately described in the following.

#### Reservoir Fabrication

**FIGURE 2:**
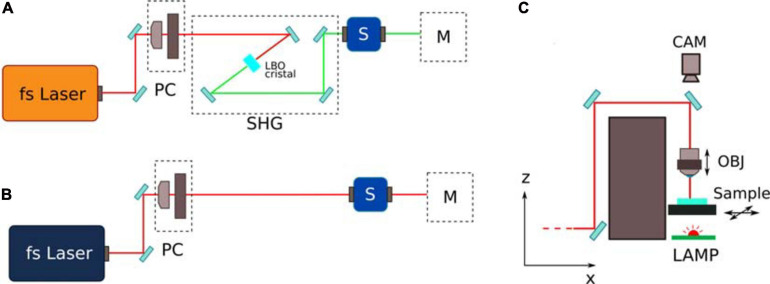
Schematic of the femtosecond laser fabrication line. **(A,B)** represent the line for the fabrication of the fused silica reservoirs and for the 2PP laser writing respectively. PC represents the power control unit, composed by a halfwave plate and by a linear polarizer. SHG represents the second harmonic generation stage, composed by a Lithium triborate (LBO) crystal with incident angle based phase matching. M represents the sample movement stage; **(C)** shows a ZX scheme of the movement stage, with a movable focusing objective (OBJ) and sample holder. A bottom led lamp and a top camera (CAM) are included for alignment and real time monitoring of the fabrication process.

The fused silica was microstructured using the second harmonic (515 nm) of a femtosecond fiber laser (Satsuma, Amplitude) operating at a repetition rate of 1 MHz, with pulse energy of 300 nJ and 230 fs duration. The laser beam was focused into the substrate using a 20 × 0.45 NA objective. The objective was mounted on a vertical translation stage, while the sample was mounted on an XY motion stage (ANT130 Series, Aerotech). In order to avoid delays between the motion stage and the laser shutter and to allow high shutter frequency, a fast-response galvo-mirror shutter was used (Altechna). The fabrication setup included a bottom illumination of the sample and a camera for sample alignment and real-time machine vision. A complete scheme of the setup is reported in Figure 2.

For the realization of the reservoirs (Figure 1A), only the external surfaces of the pools were irradiated with vertical and horizontal planes. In order to guarantee a superposition of the single laser tracks composing the planes, a vertical pitch of 10 μm and a horizontal pitch of 3 μm were chosen. The sample was then placed in a hydrofluoric acid bath at 20% volume concentration, at 35°C, for approximately 5 h to remove the modified glass (Figure 1B).

#### Multilayer Structure Realization

The photoresist film (SU-8-3025, Microchem) was deposited on a standard microscope coverslip (Menzel-Gläser, 100 μm thickness). We chose SU-8-3025 as it is known to be a biocompatible photoresist, with a good glass adhesion and a stiffness high enough to use it as a rigid environment for cells motility experiments (Liu et al., 2015).

We developed a dedicated pre-treatment procedure of the glass substrates, which guaranteed a successful realization of perfectly sealed chips made of polymerized structures embedded between them. It was first immersed overnight in chromic acid solution, then rinsed in filtered water and isopropanol and finally the surface underwent an oxygen plasma cleaning (ZEPTO, Electronic Diener).

Next, the SU8 photoresist was deposited on the surface of the coverslip and spin coated (SCS P6800) to obtain a thin film. Using about 0.9 g of resist and a maximum spin speed of 3,000 rpm, we were able to obtain a reproducible layer with thickness of 10–12 μm (Figure 1C). The three-layer structure was then assembled by gently depositing the fused silica glass on the top of the resist film (Figure 1D). The sample then underwent a soft baking at 95°C for 20 h, to reduce the solvent content in the SU8 film and reducing the thickness to 10 μm. The required baking time was much longer than simple open-top film realized with the same procedure (approximately 5 h) due to the difficulty of the solvent to evaporate once enclosed between the two glasses. Nevertheless, this passage is important to avoid bubble formation during 2PP laser writing.

#### 2PP Microchannel Constrictions Fabrication

The realization of the microchannel can be divided into two steps: the constriction irradiation and the device sealing.

The first is realized by performing 2PP through the coverglass layer in a standard reverse approach, i.e., realizing the structures flipped upside down (Figure 1E). The use of a machine vision camera allows the precise alignment of the reservoir edges with the constriction microchannel. Only the region in between the two reservoirs is irradiated, in order to reduce the overall fabrication time.

For the 2PP irradiation (see Figure 2B) we used a femtosecond fiber laser (Femtofiber pro NIR, Toptica), with repetition rate of 80 MHz, 100 fs of pulse duration and wavelength of 730 nm and we focused the beam with a 63 × 0.75 NA objective, with a compensation ring for aberration reduction (LD-plan Neofluar, Zeiss). It is important to underscore that the use of the 0.75 NA objective allowed a polymerization voxel with a cross-section diameter of ∼2 μm, compatible with the required minimum feature size, and guaranteed a reasonable fabrication time (if compared with high-NA oil-immersion objectives that require a greater number of irradiation lines due to the higher resolution). The laser irradiation parameters were optimized to obtain robust structures and a repeatable fabrication process. The constrictions were fabricated using 0.4 nJ pulse energy and a translation speed of 0.5 mm/s. Other structures, such as microchannel delimiter walls, are realized with 0.5 nJ pulse energy and a translation speed of 0.6 mm/s, due to the less strict dimension specification. With these parameters, the polymerization line has a lateral dimension in the order of 1 μm. Thus the structures are fabricated laterally by irradiating adjacent lines (shift of 1 μm) to obtain a width of 12 μm and vertically by irradiating plane by plane (shift of 1.5 μm). The overall laser writing process takes approximately 1.5 h, for a set of 18 constrictions and two edge walls to define the microchannel, with a film thickness of 10 μm.

The constriction fabrication process resolution and accuracy are governed by the voxel width (2 μm) and movement stages accuracy (500 nm), respectively. Reproducibility is governed by the reproducibility of the 2PP of SU8 which is a well-established process and, therefore, showed a high sample fabrication success rate. Nevertheless, we have not yet made a statistical study regarding this aspect.

The sealing of the device, i.e., the polymerization of the reservoir frame, was performed with a lithographic approach, irradiating the device with UV light while masking the microchannel area (Figure 1F) using a UV lamp emitting at 365 nm (Hamamatsu) with an exposure dose of 150 mJ/cm^2^ for three times. A post-baking step was performed afterward, to ensure the complete crosslinking of the SU8 polymer. The sample was placed on a hotplate at 95°C for 5 min.

The non-polymerized material was removed using a dedicated resist solvent (SU8 Developer, Microchem) with an immersion of 20 h (Figure 1G). This long developing time is necessary since the constriction area could be reached by the solvent only through the limited aperture of the two reservoirs. After this, the sample is ready for coating and biological experiments.

Different coating solutions can be applied to the chip to enhance cell adhesion without obstructing the micro-constrictions. We previously reported the successful application of the poly-L-lysine and fibronectin coating solutions (Ficorella et al., 2019). Poly-L-lysine operates by increasing the electrostatic interactions between the ions of the positively charged culture substrate and the ions of the negatively charged cell membrane. Fibronectin is an extracellular matrix protein widely expressed in multiple cell types, and abundant in connective tissues and blood. It mediates a wide range of cellular interactions with the ECM, and performs important functions in cell growth, differentiation, adhesion, and migration. Because fibronectin matrix contributes to the generation of tumor metastasis by enhancing the formation of pre-metastatic niches, the fibronectin based coating is a suitable choice in experiments with tumor cells.

### Cell Culture and Labeling

We used the adherent indifferenciated NG108-15 cell line which is a transformed hybrid cell line developed by Bernd Hamprecht in 1971 and derived from the union of rat glioma cells with cancerous mouse neuroblastoma cells in the presence of inactivated Sendai virus (Daniels and Hamprecht, 1974). NG108-15 cells have been extensively used as a model for investigating the process of neuronal differentiation in *in vitro* studies (Ingraham and Maness, 1990; Beaman-Hall and Vallano, 1993; Tojima et al., 2000; Sukma et al., 2005). The cell line was cultured in Dulbecco’s Modified Eagle’s Medium (DMEM) (Biochrom, with 4,500 mg/l glucose, L-glutamine, without sodium pyruvate) supplemented with 10% fetal calf serum FCS (Biochrom) and 1% 10,000 U/ml penicillin/streptomycin (Biochrom). For live staining of filamentous actin, 1 μM staining solution was obtained by diluting 1:1,000 1 μl of SirActin stock solution (Spirochrome) with the cell medium. 0.1 mg/ml Hoechst-34580 (Invitrogen/Molecular Probes) was used for nuclear staining. These two stains were used since the actin cortex displays the cell outline and the nucleus is a prominent structure for cell tracking.

### Migration Assays and Imaging Setup

Cells were seeded on the culture chamber of the micro-constriction chips at a density of approximately 106 cells/ml, assessed with an EVE cell counter. Regular medium was pipetted into the collecting chamber. The biochip was incubated overnight at 37°C to allow the cells to adhere to the glass bottom and initiate proliferation. Imaging experiments were begun 1 day after cell seeding in the culture reservoir. For the phase contrast long-term observations, the chips were placed in a 12-well plate with plastic bottom. The cells were observed for a maximum of 4 days, the medium was refilled every 12 h. Time-lapse phase contrast recordings with a 300 s interval between acquisitions were acquired with a 10x (NA 0.3, air) objective using a Zeiss Axio Observer Z1. The microscope was equipped with an on-stage incubation chamber that maintained the temperature at 37°C and CO_2_ concentration at 5% and with a Yokogawa CSU-X1A5000 spinning disk confocal scanning unit to acquire fluorescent images. For the fluorescent pictures, a 100x Plan-Apochromat M27 objective (NA 1.4, oil) was selected. Images were acquired with a Hamamatsu Orca Flash 4.0 camera. Gamma values were adjusted to obtain high contrast images. The device was cleaned after each experiment by vigorously pipetting mucasol detergent and ethanol (three times each, in alternate manner) and later phosphate-buffered saline (PBS) (multiple times, to ensure that no detergent residues were left in the chambers or in the microconstrictions). On average a single chip could be reused five times.

### Optical Stretcher Measurements

The size of the NG108-15 cells was measured with an optical stretcher (OS) (Gyger et al., 2014). The OS is a laser-based dual-side optical tweezer trap, generated by two optical fibers facing across a glass capillary. The cells flowing in the medium can be trapped and measured with video microscopy at their ground state in suspension, unstimulated from any adhesion effects when in contact with other cells or with the measurement device itself (Gyger et al., 2014). The trap works with a 1,064 nm Fibolux Fibotec laser source, operating with a total power impinging on the sample of 100 mW. Further details on the setup and operating principles can be found in references (Gyger et al., 2014). For these measurements, we utilized NG108-15 cells not selected for the migration assays in the biochips. Attention was paid to measure as many cells as possible (more than 1,000 cells) to obtain an accurate broad distribution of the size of each cell line.

## Results and Discussion

**FIGURE 3:**
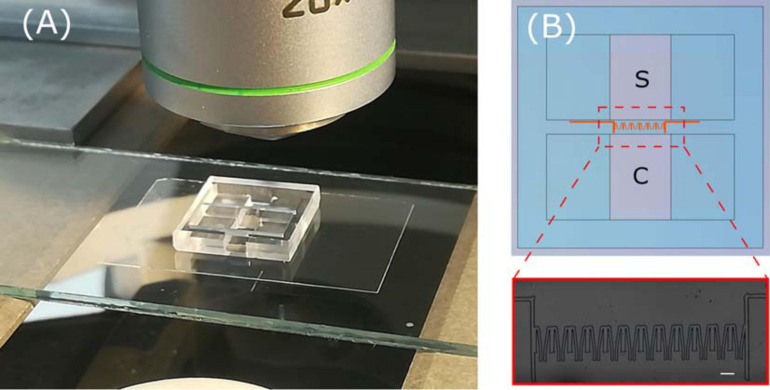
**(A)** Picture of whole device ready with blow up **(B)** of a microscope image of the constriction region. In the scheme, the sample deposition chamber and the chemoactractor chamber are labeled as S and C, respectively.

In our work we took advantage of laser writing versatility to realize devices with different topological environments. The main microfluidic chip scheme is made of an inlet chamber for cell deposition and an outlet chamber for chemoattractant deposition, each reservoir with a volume of 34 μl. The two are connected by a 2 mm wide by 400 μm long microchannel where up to 18 constrictions are hosted. The migration area easily fits into a 5× objective field of view, thus it can be used for cell tracking experiments without the need of device realignment when monitored by microscope. The scheme is shown in Figure 3, together with a picture of the overall final device.

**FIGURE 4:**
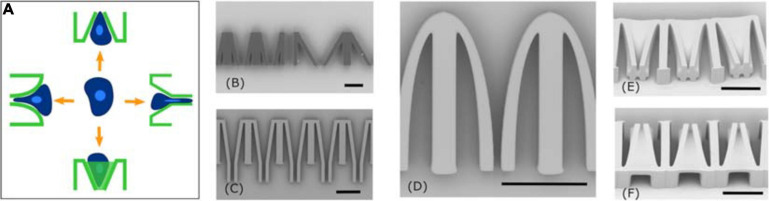
Different constriction geometries. **(A)** Scheme of four possible geometries; **(B)** simple funnel geometry realized with angles of 12 and 30 degrees; **(C)** funnel geometry combined with 40 μm long tunnels; **(D)** close up of single constriction with elliptical profile; **(E,F)** 3D funnel geometry. Scale bars corresponds to 50 μm.

The constriction region geometry can be easily reorganized depending on the experimental cell migration behavior under study (see Figure 4A). The first geometry is a funnel like structure with an aperture that linearly diminishes, at a constant angle, from a few tens of micrometers to a set value in the range of 3–7 μm. The angle, as well as the minimum aperture, can be chosen depending on the application. For instance, structures with different angles of 12 and 30° are shown in Figure 4B. Inside these passages, the migrating cell is subjected, at least from a geometrical perspective, to pure compressive stress exerted by both lateral walls and once it reaches and passes the constriction it arrives to a free space. These types of structures were already successfully used for the study of mammalian cancer cell invasiveness (Ficorella et al., 2019), showing that during their traveling through the funnel the cells occupy the whole empty surface (until walls) and transit with an amoeboid-like motility mode.

Alternative funnel geometries can be easily designed and fabricated with our approach, with some examples shown in Figures 4C–F. Each of these can simulate other interactions between the cells and the microenvironment.

It is possible to induce a variable confinement to the migrating cell also vertically, by adding an inclined roof to the constriction channel (Figures 4E,F). In this geometry the transversal profile of the constriction aperture is variable, going from ∼100 μm^2^ to a square area of 5 μm by 5 μm. In this structure the cell will be subjected to a variable constrain from the top and lateral walls during the migration.

We can also control the geometry of the constriction wall, for example, by following an elliptical arc fashion, creating a funnel with a width that diminishes in a non-linear profile (Figure 4D). If compared with the previous constrictions this kind of topology should induce a different deformation of the cell during migration. Additionally, the direction of shear stress between walls and cell membrane changes during cell squeezing and might influence the cell migration behavior.

All the previous geometries are based on an immediate exit and relaxation of the cell into a non-confined chamber after the narrow constraint. In order to study the influence of a long passage through a constriction on the migrating cells, such as molecular adaption and memory effects through transient crosslinking and sacrificial bond breaking, we introduce an appended micro-tunnel after the funnel structure, with the same minimum dimension of the constriction and with a length much greater than the cell diameter (i.e., 40 μm) as shown in Figure 4C. This geometry, whose length can be tailored depending on the cell type under study, will illustrate how long the cells can maintain their migration regime inside the micro-tunnel (i.e., amoeboid one) and if the long transition time could train their motility properties or influence their viability afterward.

We have used this last topology as a proof of principle to demonstrate the cell viability of our murine neuronal cancer cells, NG108-15, under high physical confinement.

NG108-15 is a transformed hybrid cell line developed by Bernd Hamprecht in 1971 and derived from the union of rat glioma cells with mouse neuroblastoma cells in the presence of inactivated Sendai virus (Daniels and Hamprecht, 1974). Glioma is a neoplasia that originates from glial cells, and infiltrates into the surrounding brain tissue. It is the most common type of cancer of the central nervous system and is characterized by a broad spectrum of biological behaviors (Mesfin and Al-Dhahir, 2020). Neuroblastoma is a malignant undifferentiated extra-cranial tumor originating from paraspinal sites near the sympathetic nervous system, and it is most commonly observed in children of 5 years of age or younger (Bahmad et al., 2019). Neuroblastoma cells used to develop the NG108-15 cell line were specifically derived from ganglion cells near the spinal cord.

Given the intrinsically invasive nature of glioma and neuroblastoma, it can be assumed that neuronal neoplastic cells must possess the ability to infiltrate dense tissue. Although the NG108-15 cell line has been previously employed in the past for cell migration studies (Kilian et al., 2008; Jacobshagen et al., 2014; Abe et al., 2018; Fendler et al., 2019), it is not known yet how they would react to migration through strict confinement. Specifically, it has not been shown whether the whole cell body has the ability to squeeze through very tight spaces, or whether this can be achieved only by its dendritic protrusions. This prompted us to choose this specific cell line for the migration assay presented here.

**FIGURE 5:**
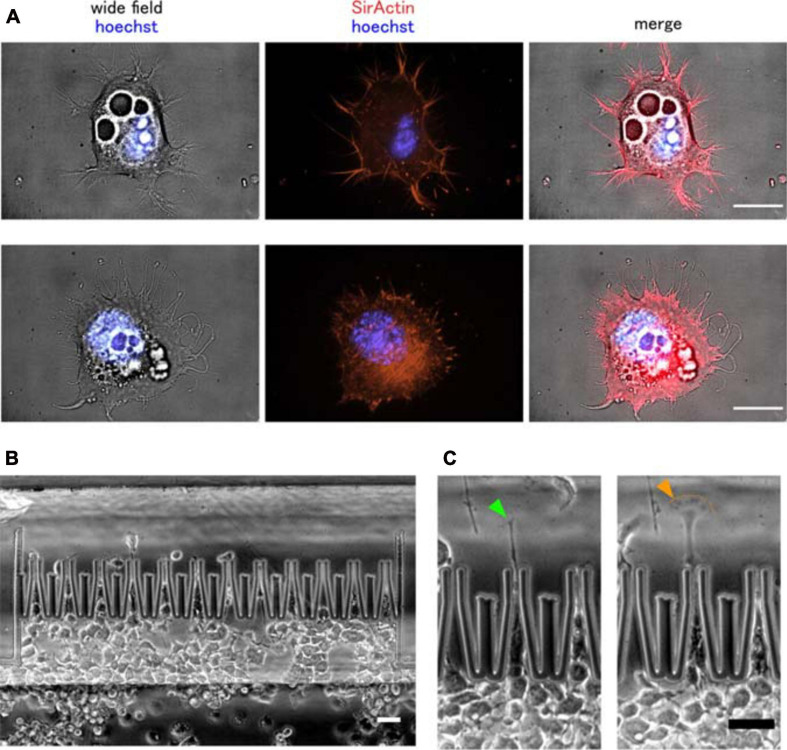
Murine neuronal cancer cell migration through narrow micro-constrictions. **(A)** Wide field and fluorescent images of two NG108-15 cells. These cells grown in 2D culture exhibit spontaneous, microtubule-driven neurite growth. Importantly, actin can still be found within the neurite protrusions, not only at their tips. Actin filaments are shown in red, nuclei are shown in blue. Scale bar is 20 μm. **(B)** Overview of the cell migration into the chip, **(C)** growth of one main neurite protrusion was observed in NG108-15 cells actively migrating through the micro-constrictions. The cell neurite exhibited a dynamical behavior during the squeezing phase, with its growth cone morphology alternating multiple times between a tight, thread-like configuration (green arrow) and a more flattened, lamellipodial-like configuration (orange arrow). Scale bar is 50 μm.

In 2D culture, NG108-15 cells are characterized by a spread-out, fibroblast-like morphology and spontaneous growth of short neurites (dendrites) (see Figure 5A). Neurites are thin and dynamic, microtubule-based protrusions extended to at least the dimension of the cell body. Neurites undergoing active elongation usually contain a filamentous actin rich growth cone. In neurons one of the neurites polarizes and lengthens even further to form an axon. Shorter neurites (dendrites) receive incoming cues, whereas the axon is capable of propagating signals over long distances. Neuronal polarization requires a rupture of cellular symmetry in order to allow neurite selection and determine the orientation of the neurite growth in a way to allow for a correct signal flow in a specific neuronal network (Heine et al., 2015; Richter and de Anda, 2015).

The OS measurements gave an average diameter of the NG108-15 cell body of ∼ 15 μm (cell radius = 7,518 μm) (see Supplementary Figure 4). Therefore we fabricated microfluidic chips containing 12 constriction tunnels, each with a width between 3 and 7 μm which is considerably smaller than the overall cell size.

In the fluidic chips, the NG108-15 cells displayed motile behavior and were able to survive the compressive stress generated by the hard channel walls (data shown in Supplementary Videos 1, 2). It is important to notice that the motile behavior was not limited to the neurites, but involved the whole cell body. No chemoattractant was necessary to stimulate directional cell migration toward the collecting chamber. As an individual cell enters a constriction, its motion undergoes a temporary arrest due to the compressive stress caused by the narrowing funnel-like geometry of the constriction, and starts a back-and-forth motion to force its entrance (see Supplementary Video 2). While whole cells were trying to squeeze through the micro-channels, we observed the formation of a main neurite at the cell front. The neurite exhibited a very dynamic behavior, alternating between periods of elongation, retraction, turning and branching as typical for growth cone motion. The time needed for individual cells to squeeze through a constriction spans several hours (approximately 15 h for the cell shown in Supplementary Video 2, migrating in the central constriction). In this assay, it is legitimate to assume that neurite initiation and elongation were mainly determined by the spatial cues induced by the geometry of the constriction.

We previously showed (Ficorella et al., 2019) how actin stress fibers formation is needed for motile human breast carcinoma cells to actively depolymerize their actin cytoskeleton and achieve migration through the micro-funnels. Fluorescent live cell imaging of filamentous actin in NG108-15 cells suggests that when they were fully adhering to the 2D glass surface and engaged in neurite growth, actin was primarily distributed along the cortex beneath the cell membrane and within the neurites themselves, with no evidence of actin stress fibers formation within the cell body (see Figure 5A). Actin fibers, however, were clearly visible in cells that were weakly adhering to the surface, maintained a rounded morphology and were characterized by little to no neurite formation (Supplementary Video 3), thus suggesting that NG108-15 cells are able to engage in stress fiber-based contractility in situations of low adhesion.

Generally, neuronal growth cones located at the neurite tip are built on distinct actin-rich structures, such as lamellipodia and filopodia, both located at the periphery of the growth cone. Lamellipodia are the easiest to detect, having a spread-out and flattened morphology containing a meshwork of actin filaments, whereas actin inside filopodia is organized into tightly packed and parallel bundles (Dehmelt and Halpain, 2004). During NG108-15 migration abrupt extension, collapse and interchange between lamellipodia and filopodia-like protrusions at the neurite tip was observed, when the neurite was pushed beyond the micro-channel aperture to probe the environment and initiate migration of the whole cell body (see Figures 5B,C).

It is important to notice that for each experiment only a limited number of cells are clearly observed to actively migrate through the constriction row as single cells (see Figure 5B). Once the culture reservoir reaches high confluence, the adherent cells begin to cover the surface of the constriction region and crowd in the constrictions, making it impossible to distinguish actively migrating single cells. This sets an important limitation in the use of our biochips, since multiple assays are necessary in order to gather enough data for statistical analysis. We are studying new device configurations that will help to increase the number of migrating events, in order to allow for a statistical analysis in each experiment. In the case of the NG108-15 cell line, cell proliferation and migration, as well as neurite formation, arrested when cells reached maximum confluence in the culture chamber (see Supplementary Video 1). This suggests that having enough space for neurite outgrowth is essential to initiate migration of the whole cell body in neuronal cells in situations of strong confinement, especially when cell size considerably exceeds the confining pores.

For the first time, the highly invasive nature of hybrid neuronal cancer cells is shown in a migration assay where the neuronal cells as a whole are undergoing relocation after achieving migration through a highly compressive and rigid environment. The actin-based growth cone drove growth of the neurite through the funnels, by means of processes driven by actin polarization and depolymerization. An important role during cell migration through the narrow pores was likely played by the microtubule (MT) scaffold built in the neurite protrusions. Microtubules are the most important cytoskeletal component of neurons, as all neurites contain a MT-based core, and the presence of MTs is what factually distinguishes neurites from filopodia or other actin-based protrusions. Therefore, it is legitimate to assume that microtubule-driven neurite outgrowth is necessary for migration of a neuronal cell body through narrow spaces, although more studies need to be carried out in this respect.

## Conclusion

We presented a hybrid approach, using femtosecond laser micromachining, for the fabrication of microfluidic chips for cell migration studies. It consists in the realization of a fused silica frame with millimetric-sized features and of a multilayer structure with a film of polymeric resist and optical quality cover glass. Inside the film the microfluidic channel, containing custom-shaped micro-constrictions, was realized. The presence of two reservoirs in the fused silica frame provides an easy user interface. At the same time the cover glass acts as a visualization window, guaranteeing good optical access and a minimum of optical aberrations.

The geometry of the migration environment topology can be easily adapted to the needs of the specific cell line in analysis, thanks to the fast prototyping capability of 2PP. In our work we presented four possible geometries that can be used to probe cells with different mechanical properties during their motion. A single constriction has a lateral size of about 100 μm, thus tens of them can be placed side by side, depending on the experimental field of view, parallelizing the analysis.

As a proof of concept, we have used one of these designs to characterize the migration behavior of NG108-15 cells. For the first time, the highly invasive nature of these cells is shown in a migration assay through narrow constrictions.

This result shows how our device can be used to address specific problems and enhance the capacity to analyze cell migration dynamics. Moreover, thanks to the fabrication technique, new and more complex construction geometries can be designed, tailoring the device to the biological issue under study.

## Data Availability Statement

The raw data supporting the conclusions of this article will be made available by the authors, without undue reservation.

## Author Contributions

JK and RO designed the research. CF performed the micro-constriction experiments. HE performed the OS measurements. FS and RM designed and realized the devices. FS, RM, and CF wrote the manuscript. All authors contributed to the article and approved the submitted version.

## Conflict of Interest

The authors declare that the research was conducted in the absence of any commercial or financial relationships that could be construed as a potential conflict of interest.
