# Supplementary material for: Rapid Prototyping of 3D Biochips for Cell Motility Studies Using Two-Photon Polymerization
Source: Front Bioeng Biotechnol. 2021 Apr 13;9:664094. doi: 10.3389/fbioe.2021.664094 (PMC8078855; doi:10.3389/fbioe.2021.664094)
Supplement: Supplementary file 1 [file Data_Sheet_1.pdf]

Supplementary Material

1 Supplementary Figures and Tables

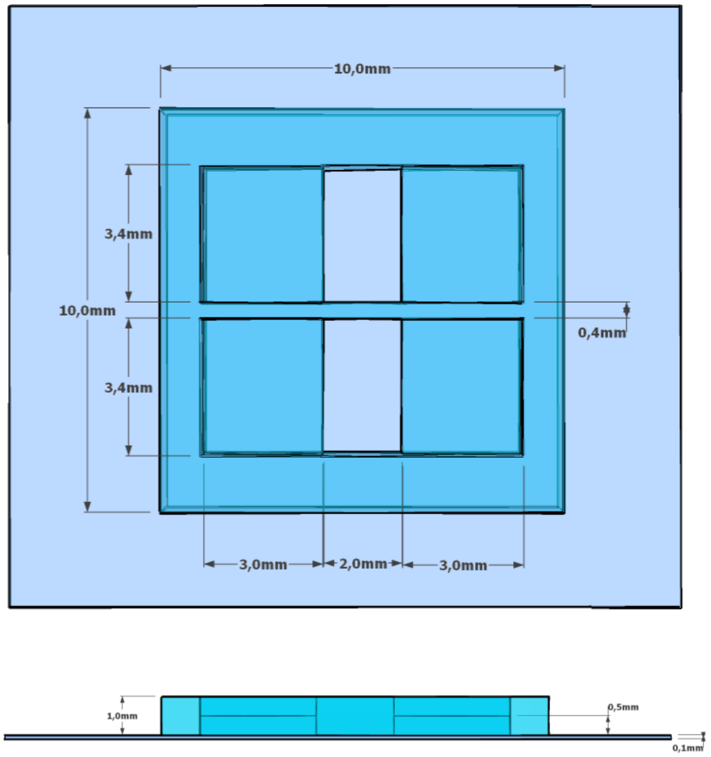

Supplementary Figure 1. Scheme of the device design. Measures of fused silica microstructured substrate and cover glass thickness are reported.

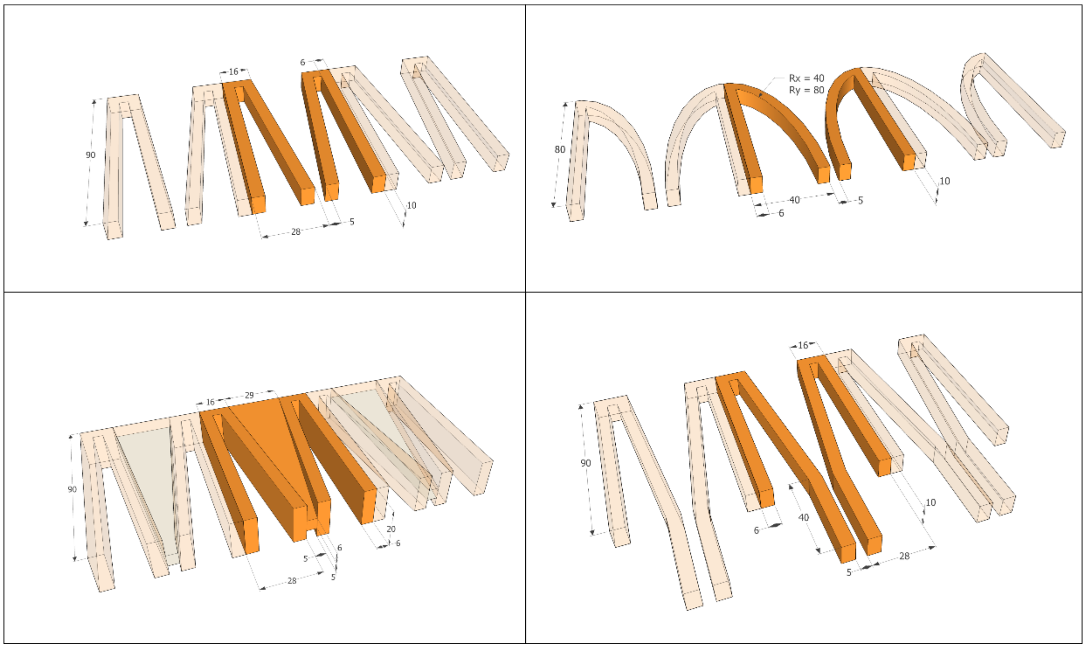

Supplementary Figure 2. Three-dimensional schematic of the constrictions geometries. As an example, only three replicas of constriction are reported in each subfigure. The reported dimensions correspond to the ones used in our work, but thanks to the mask less approach of 2PP technique, these dimensions can be easily changed. Clockwise: simple constriction, elliptical profile constriction, simple constriction with tunnel extension, 3D constriction with inclined rooftop.

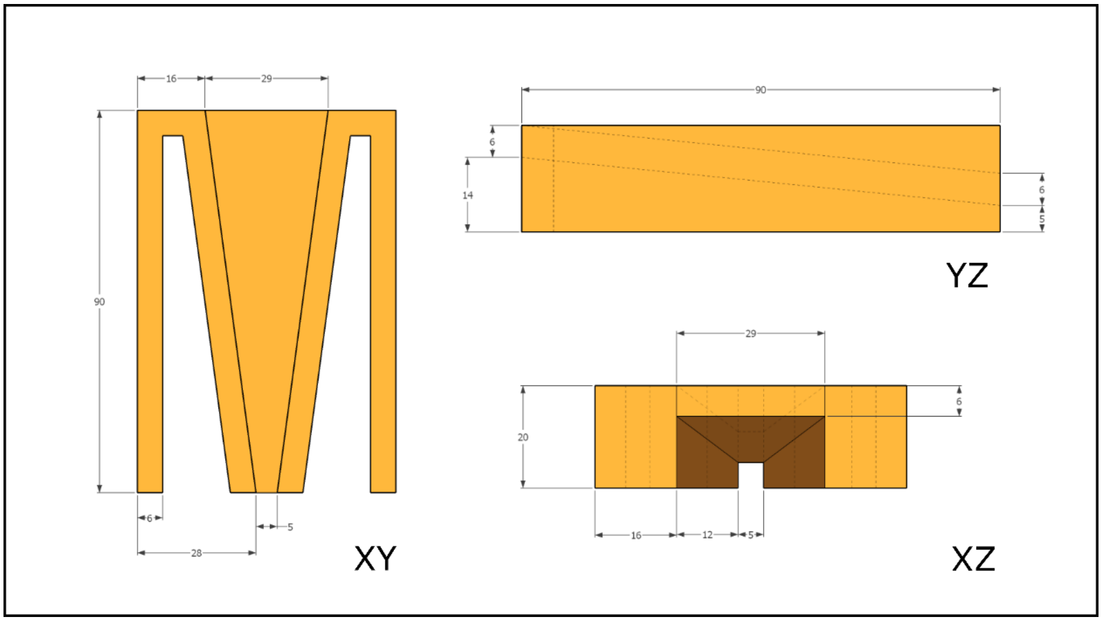

Supplementary Figure 3. Orthogonal projections of 3D constriction with inclined rooftop (see Figure S2 for three-dimensional representation).

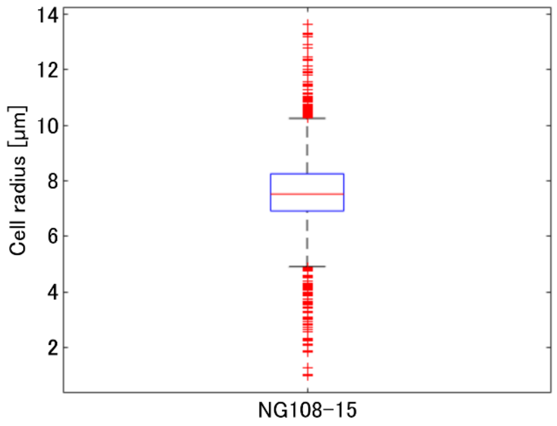

Supplementary Figure 4. Box-plot of average cell radius of NG108-15 cells (2039 cells), as measured with Optical Stretcher.

2 Supplementary Video

Supplementary Video 1. Murine neuronal cancer cell migration in micro-constriction chip. Phase contrast time lapse imaging of NG108-15 cells migrating on an untreated chip. Cells are observed to extend an axon protrusion in the direction of and through the channel opening, which has the ability to generate enough traction to force migration of the whole cell body through the micro-constrictions. Migration stops as the cells reach maximum confluence in the culture chamber (15 fps, each frame representing 5 min.).

Supplementary Video 2. Detail of murine neuronal cancer cell migration in micro-constriction chip. Closer view of NG108-15 cells successfully squeezing through the narrow channels (detail from Supplemental video 1). The width of the constriction is 5 µm. (15 fps, each frame representing 5 min.).

Supplementary Video 3. Actin filament structure of NG108-15 cell in 2D culture. Segmentation along the z-axis showing the fluorescent live cell labeling of the filamentous actin cytoskeleton of an NG108-15 cell. The cell is characterized by a rounded morphology, as adhesion to the glass surface is likely weak in this case, and clear formation of parallel actin stress fibers within the cell body. Actin is shown in red, nucleus in blue.
